# Validity and reliability of a pediatric patient classification instrument^1^

**DOI:** 10.1590/0104-1169.3575.2457

**Published:** 2014

**Authors:** Ariane Polidoro Dini, Daniela Fernanda dos Santos Alves, Henrique Ceretta Oliveira, Edinêis de Brito Guirardello

**Affiliations:** 2 PhD, RN, Hospital de Clínicas, Universidade Estadual de Campinas, Campinas, SP, Brazil; 3 Doctoral student, Faculdade de Enfermagem, Universidade Estadual de Campinas, Campinas, SP, Brazil; 4 Statistician, Faculdade de Enfermagem, Universidade Estadual de Campinas, Campinas, SP, Brazil; 5 PhD, Associate Professor, Faculdade de Enfermagem, Universidade Estadual de Campinas, Campinas, SP, Brazil

**Keywords:** Health Evaluation, Pediatric Nursing, Validation Studies, Workload

## Abstract

**OBJECTIVES:**

to assess the construct validity and reliability of the Pediatric Patient Classification Instrument.

**METHODS:**

correlation study developed at a teaching hospital. The classification involved 227 patients, using the pediatric patient classification instrument. The construct validity was assessed through the factor analysis approach and reliability through internal consistency.

**RESULTS:**

the Exploratory Factor Analysis identified three constructs with 67.5% of variance explanation and, in the reliability assessment, the following Cronbach's alpha coefficients were found: 0.92 for the instrument as a whole; 0.88 for the Patient domain; 0.81 for the Family domain; 0.44 for the Therapeutic procedures domain.

**CONCLUSIONS:**

the instrument evidenced its construct validity and reliability, and these analyses indicate the feasibility of the instrument. The validation of the Pediatric Patient Classification Instrument still represents a challenge, due to its relevance for a closer look at pediatric nursing care and management. Further research should be considered to explore its dimensionality and content validity.

## Introduction

The use of patient classification instrument permits characterization inpatient units, estimating the nursing workload, supporting staff dimensioning, identifying changes in patients' care needs, promoting improvements in team competency and involvement, besides being an objective and practical method to obtain information and statistical data^(^ 1 ^-^ 3 ^)^.

In daily practice, it can be observed that patients are classified intuitively through task division, which does not always reflect their care needs. A changed perspective, from the number of tasks that are to be performed to care planning focused on the patients' needs, can expand the possibilities of nursing's health promotion activities and also improve the satisfaction and involvement with the work outcomes. In that sense, it is important to use specific instruments for each clientele.

The Pediatric Patient Classification Instrument (PPCI)^(^ 4 ^)^ permits classifying pediatric patients in five care categories: Minimal, Intermediary, High dependence, Semi-intensive and Intensive^(^ 5 ^)^. The factor evaluation instrument consists of 11 indicators, composed of four situations of care dependence, scored from one to four points, increasing with the level of care demands.

Validity and reliability are crucial aspects in the use of an instrument, as the validity is related to its precision and the reliability is the instrument's ability to present accurate measures. The validity can be assessed, among other aspects, with regard to the content and the construct. The content validity refers to the dimensions of the instrument domain, their conceptual definition, readability and clarity; the construct validity presupposes that the instrument measures a theoretical construct and aims to validate the theory underlying the measure. The reliability can be assessed with regard to the homogeneity, or correlation between each question in a scale and another question in the same scale; and with regard to the equivalence, measured by the agreement between two evaluators' measures when the instrument is applied at the same time^(^ 6 ^)^.

In the development process of the PPCI, the content validity analysis by experts was performed by means of the Delphi technique and the inter-rater reliability was verified^(^ 4 ^)^. As the PPCI is used to support management decisions at pediatric units, its validation process cannot be impervious and demands successive studies to monitor its validity and reliability. This study intends to assess the construct validity and reliability of the PPCI.

## Methods

This correlation study was undertaken at a pediatric unit of a teaching hospital in the interior of the State of São Paulo, which consists of 58 inpatient beds and ten intensive care beds.

Approval for the study was obtained from the Research Ethics Committee (Process 646/2010). The signing of the Informed Consent Form was waived as the use of the PPCI is inherent in the nursing work process and the application of the instrument did not involve submitting the patients to any procedure.

The sample consisted of 227 pediatric patients hospitalized between September 2011 and June 2012. Two of the authors collected the data with the help of a registration worksheet, including information about age, sex, reason for hospitalization and classification of each patient according to the PPCI.

The PPCI consists of 11 care indicators: Activity, Physiological controls assessment, Drug therapy, Oxygenation, Cutaneous and Mucosal Integrity, Mobility and ambulation, Personal hygiene, Feeding and hydration, Eliminations, Participation of the accompanying person and Support network. Each indicator is assessed with the help of four situations, scored in rising order according to the care demand. The sum of the scores permits classifying the patient in one of the five care categories established in the literature: Minimal (11-17 points), Intermediary (18-23 points), High dependence (24-30 points), Semi-intensive (31-36 points) or Intensive (37-44 points)^(^ 4 ^-^ 5 ^)^.

The data were organized in an electronic worksheet in Microsoft Excel^(r)^ and analyzed using SPSS 20.0^(r)^ for Windows. The construct validity was assessed through factor analysis, applying the Exploratory Factor Analysis (EFA) technique. All variables were ordinal and the factor extraction method chosen was the principal component analysis with orthogonal Varimax rotation. An index of 20 patients per PPCI indicator was considered, higher than the methodological recommendation of five patients per indicator, as it is emphasized in the literature that, the larger the sample, the more reliable the EFA will be^(^ 6 ^-^ 7 ^)^.

The Kaiser-Meyer-Olkin (KMO) and Bartlett's Sphericity tests were performed to verify the data adjustment to the EFA. The KMO coefficients show the extent of the variance the indicators have in common, in which coefficients between 0.6 and 0.7 are considered reasonable; between 0.7 and 0.8 medium; between 0.8 and 0.9 good and superior to 0.9 very good. Bartlett's sphericity test is based on the statistic distribution of Chi squared and, with a view to the appropriateness of the factor analysis method, the null hypothesis about the identity of the correlation matrix should be rejected, that is, the significance of Bartlett's sphericity test should be inferior to 0.05^(^ 6 ^-^ 7 ^)^. The construct validity analysis according to the EFA is guaranteed when the total variance explanation represents more than 60% and, according to the Kaiser criterion, factors should be extracted with an Eigenvalue superior to one in order to identify the construct domains^(^ 6 ^-^ 7 ^)^.

The commonalities represent the extent of the variance explanation of each indicator based on the factors identified. For the indicator to be representative, its commonality index should be superior to 0.6^(^ 6 ^-^ 7 ^)^. The factor loadings represent the correlation between the indicator and the extracted factor. Thus, coefficients between 0.30 and 0.40 are considered minimal; factor loadings between 0.50 and 0.70 are significant and loadings superior to 0.70 indicate a well-defined structure, which is the target of any factor analysis^(^ 6 ^-^ 7 ^)^. The residues represent the aspects of the variance the indicators do not explain, and a percentage of more than 50% of residues superior to 0.05 is not desirable^(^ 6 ^-^ 7 ^)^.

The reliability of the PPCI was assessed by means of the internal consistency with three parameters: item-total correlations, inter-item correlations and Cronbach's alpha (α). For the PPCI to be considered reliable, the item-total correlation should be superior to 0.50; the inter-item correlations should be super to 0.30 and Cronbach's alpha superior to 0.70^(^ 6 ^,^ 8 ^)^.

## Results

The sample characteristics in terms of sex, age and reason for hospitalization are displayed in Table 1. The sample mostly included patients between one and six years of age, male, predominantly hospitalized due to surgical procedures or respiratory conditions.

**Table 1:** Sample characteristics (N=227). Campinas, SP, Brazil, 2013

Variables		n	%
Age range (years)			
	<1	64	28.0
	1 to 6	77	34.0
	7 to 11	47	21.0
	12 to 17	30	13.0
	≥18	9	4.0
Sex			
	Male	136	59.9
	Female	91	40.1
Reason for hospitalization			
	Surgical procedures	50	22.0
	Respiratory conditions	49	21.5
	Genital-urinary conditions	27	11.9
	Clinical conditions*	25	11.0
	Neurological conditions	24	10.6
	Infections	21	9.3
	Liver or gastrointestinal tract conditions	14	6.2
	Other reasons^ †^	17	7.5

* Rheumatic, dermatologic conditions, immunodeficiency, dehydration, malnutrition, cardiac disease †Orthopedic conditions, diagnostic procedures and accidents

As regards the classification in care demand categories, most patients were classified as intermediary (30%) or high dependence (28.6%) (Table 2).

**Table 2:** Patient classification according to Pediatric Patient Classification Instrument (PPCI) care categories (N=227). Campinas, SP, Brazil, 2013

Care Category	<1 year			1-6 years			7-11 years			12-17 years			≥18 years			Total	
	n	%		n	%		n	%		n	%		n	%		n	%
Minimal	-	-		3	3.9		3	6.4		13	43.3		1	11.1		20	8.8
Intermediary	6	9.4		28	36.4		18	38.3		12	40.0		4	44.5		68	30.0
High dependence	22	34.4		22	28.6		13	27.7		5	16.7		3	33.3		65	28.6
Semi-intensive	10	15.6		17	22.1		9	19.1		-	-		1	11.1		37	16.3
Intensive	26	40.6		7	9.0		4	8.5		-	-		-	-		37	16.3
Total	64	100		77	100		47	100		30	100		9	100		227	100

In the assessment of the construct validity through exploratory factor analysis, three factors were extracted from the PPCI construct, with 67.5% of variance explanation, representing the three care domains. The Patient domain represented 32.6% of the variance, the Family domain 22.3% and the Therapeutic procedures domain 12.6% of the variance explanation. The principal component extraction method found 52.0% of residues with coefficients >0.05. The KMO coefficients, communalities and factor loading of each indicator per extracted domain are displayed in Table 3.

**Table 3:** Construct analysis of the Pediatric Patient Classification Instrument (PPCI) (N=227). Campinas, SP, Brasil, 2013

Construct		KMO*	Communality	Factor loading
Domain: Patient				
	Personal hygiene	0.85	0.80	0.86
	Feeding and hydration	0.89	0.67	0.81
	Mobility and ambulation	0.87	0.74	0.77
	Activity	0.89	0.73	0.69
	Eliminations	0.85	0.58	0.65
	Oxygenation	0.89	0.73	0.65
Domain: Family				
	Support network	0.79	0.79	0.87
	Participation of the accompanying person	0.82	0.73	0.82
Domain: Therapeutic procedures				
	Drug therapy	0.68	0.77	0.87
	Cutaneous-mucous integrity	0.86	0.36	0.50
	Physiological controls assessment	0.88	0.52	0.39

* Kaiser-Mayer-Olkin test

In the reliability assessment, the following Cronbach's alpha coefficients were found: 0.92 for the instrument as a whole; 0.88 for the Patient domain; 0.81 for the Family domain; 0.44 for the Therapeutic procedures domain.

For the internal consistency assessment of the PPCI, the inter-item and item-total correlation coefficients are shown in Table 4.

**Table 4:** Item-item and item-total correlation of Pediatric Patient Classification Instrument (PPCI) (N=227). Campinas, SP, Brazil, 2013

Indicator*	I-1	I-2	I-3	I-4	I-5	I-6	I-7	I-8	I-9	I-10	I-11
I-1											
I-2	0.43										
I-3	0.74	0.56									
I-4	0.11	0.33	0.15								
I-5	0.26	0.16	0.23	0.21							
I-6	0.60	0.41	0.57	0.11	0.16						
I-7	0.29	0.34	0.26	0.16	0.20	0.41					
I-8	0.61	0.45	0.59	0.15	0.29	0.65	0.57				
I-9	0.68	0.48	0.67	0.11	0.34	0.54	0.43	0.77			
I-10	0.42	0.41	0.45	0.12	0.29	0.24	0.18	0.33	0.44		
I-11	0.48	0.44	0.52	0.11	0.26	0.24	0.13	0.31	0.39	0.69	
Total	0.80	0.69	0.82	0.29	0.44	0.69	0.52	0.79	0.83	0.65	0.65

* I-1: Activity; I-2: Physiological controls assessment; I-3: Oxygenation; I-4: Drug therapy; I-5: Cutaneous and mucosal integrity; I-6: Feeding and hydration; I-7 Eliminations; I-8: Personal hygiene; I-9: Mobility and ambulation I-10: Participation of the accompanying person; I-11: Support network

## Discussion

The classification of patients under six years of age in the minimal or intermediary care category is not considered appropriate for the definition of the care categories, considering that the number of nursing care hours established by the Federal Nursing Council, corresponding to only 3.8 hours for minimal care and 5.6 hours for intermediary care, do not reflect the actual care needs of pediatric patients under six years of age^(^ 5 ^,^ 9 ^)^.

Bartlett's sphericity test indicated that the analyzed data adjust to the EFA and the sample adequacy test, with KMO coefficients that are considered very good for nine indicators, average for Support network and reasonable for Drug therapy, indicating that the EFA results can be generalized and that the variance proportion of the PPCI indicators share a construct^(^ 6 ^-^ 7 ^)^.

Based on the EFA, it was verified that the PPCI covers three pediatric nursing care domains: family, patient and therapeutic procedures and, as a factor assessment instrument, its validity does not relate to the number of indicators or situations it covers, but to its concept as a whole, as each indicator of the instrument represents a list of potential care needs^(^ 10 ^-^ 11 ^)^.

Each instrument domain does not represent a sum of individual care tasks or procedures, but nursing values based on the notion that the patient needs are multidimensional and depend on the complex objective and subjective interactions^(^ 12 ^)^.

According to the established criteria, the extraction of three domains represents a care model centered on the child and his/her family, whose care approach presupposes the consideration of the domains that result in the child's health condition: the sick biological body; the child's mental, spiritual and social dimensions; and the family, seen holistically, as responsible for the healthcare shared with the professionals during the hospitalization^(^ 10 ^-^ 11 ^,^ 13 ^)^.

The three resulting domains underline the importance of the accomplishment of pediatric nursing interventions inextricably from health promotion, disease prevention, health recovery and rehabilitation, in which it is fundamental to take into account the child and family's singularities with a view to qualified and humanized healthcare^(^ 13 ^)^.

The presence of more than 50% of residues with coefficients superior to 0.05 and the communality coefficients for the indicators Physiological controls assessment, Cutaneous and mucosal integrity and Eliminations suggested that these indicators could not be considered representative in their respective constructs and, although the exclusion of these indicators may be considered in the literature^(^ 6 ^-^ 7 ^)^, developing new studies with interventions in the content of these indicators seems to be more appropriate to improve the clarity of the instrument contents.

The residues represent the aspects of the variance the indicators do not explain^(^ 7 ^)^. It would be desirable for the residue counts with coefficients superior to 0.05 to be present in less than 50% of the data, which reveals the need for research about the clarity of the instrument contents.

A well-defined structure was evidenced for the indicators Personal hygiene, Feeding and hydration, Mobility and ambulation, Support network, Participation of the accompanying person and Drug therapy, with factor loadings superior to 0.70. The indicators Activity, Eliminations, Oxygenation and Cutaneous mucous integrity showed significant factor loadings between 0.50 and 0.69. The indicator Physiological controls assessment showed a minimum interpretation level of the PPCI, with a factor loading of 0.39, which suggests the need to review its content validity.

The reliability of the PPCI was evidenced through a Cronbach's alpha coefficient superior to 0.75^(^ 8 ^)^ for the instrument as a whole and for the Patient and Family domains; as well as by the item-total correlation coefficients superior to 0.50 and inter-item correlation coefficients superior to 0.30 between the indicators in these domains.

As regards the indicators Cutaneous and mucosal integrity and Drug therapy in the Therapeutic procedures domain, with a Cronbach's alpha coefficient of 0.44; inter-item correlations inferior to 0.30; and item-total correlations inferior to 0.50, it is highlighted that this apparent lack of reliability can be interpreted by the fact that the Therapeutic procedures domains comprise indicators of different tasks during the hospitalizations and refer to tasks focused on the disease, while the other indicators are focused on the conditions of the pediatric patients and their relatives.

The analyses indicate the feasibility of patient classification through the PPCI, but suggest further research to confirm the three domains identified in the EFA, as well as to review the content validity of the instrument to investigate whether clarity, pertinence or relevance problems caused low factor loadings or the presence of residues superior to 50%.

## Conclusion

The construct validity of the PPCI can be proven by the variance explanation superior to 60% in the three domains: Family, Patient and Therapeutic procedures, as well as the factor loadings superior to 0.30 and appropriate coefficients for the other indices that were calculated.

The Cronbach's alpha coefficients superior to 0.70 for the instrument as a whole and for the Family and Patient domains, as well as the correlations superior to 0.50 between the indicators and the total and superior to 0.30 between the indicators of each instrument domain evidenced the reliability of the PPCI.

The validation of the PPCI is a pediatric nursing management resource in attempts to balance the care demand and supply. In addition, the application of the instrument drives clinical nursing assessment towards care delivery that is not only focused on the disease, tasks and therapeutic procedures, but also inspires the assessment of family members and patients, looking at their care needs, and can recover a reference to the range of nursing work.

Instruments like the PPCI are scarce in the literature. Therefore, its validation remains a challenge and, in view of its relevance for a more sophisticated look on pediatric nursing care and management, further research is needed to re-explore its dimensionality and content validity.
